# Supplementary material for: Current Siberian heating is unprecedented during the past seven millennia
Source: Nat Commun. 2022 Aug 25;13:4968. doi: 10.1038/s41467-022-32629-x (PMC9411110; doi:10.1038/s41467-022-32629-x)
Supplement: Supplementary file 1 — Supplementary Information [file 41467_2022_32629_MOESM1_ESM.pdf]

SUPPLEMENTARY INFORMATION FOR

Current Siberian heating is unprecedented during the past seven millennia

Rashit M. Hantemirov[1,2], Christophe Corona[3,4,5], Sebastien Guillet[3], Stepan G. Shiyatov†[1], Markus Stoffel[3,5,6], Timothy J. Osborn[7], Thomas M. Melvin[7], Ludmila A. Gorlanova[1], Vladimir V. Kukarskih[1,2], Alexander Y. Surkov[1], Georg von Arx[8,9], Patrick Fonti[8]

[1]Institute of Plant and Animal Ecology, Ural Division of the Russian Academy of Sciences, Ekaterinburg 620144, Russia. [2]Ural Federal University, Ekaterinburg 620002, Russia. [3]Climate Change Impacts and Risks in the Anthropocene (C-CIA), Institute for Environmental Sciences, University of Geneva, 1205, Geneva, Switzerland. [4]Geolab, UMR 6042 CNRS, Université Clermont Auvergne, F-63057 Clermont-Ferrand, France. [5]Department F.A. Forel for Environmental and Aquatic Research, University of Geneva, 1205, Geneva, Switzerland. [6]Department of Earth Sciences, University of Geneva, 1205, Geneva, Switzerland. [7]Climatic Research Unit, School of Environmental Sciences, University of East Anglia, Norwich NR4 7TJ, UK. [8]Swiss Federal Research Institute WSL, 8903 Birmensdorf, Switzerland. [9]Oeschger Centre for Climate Change Research, University of Bern, 3012, Bern, Switzerland

SUPPLEMENTARY FIGURES

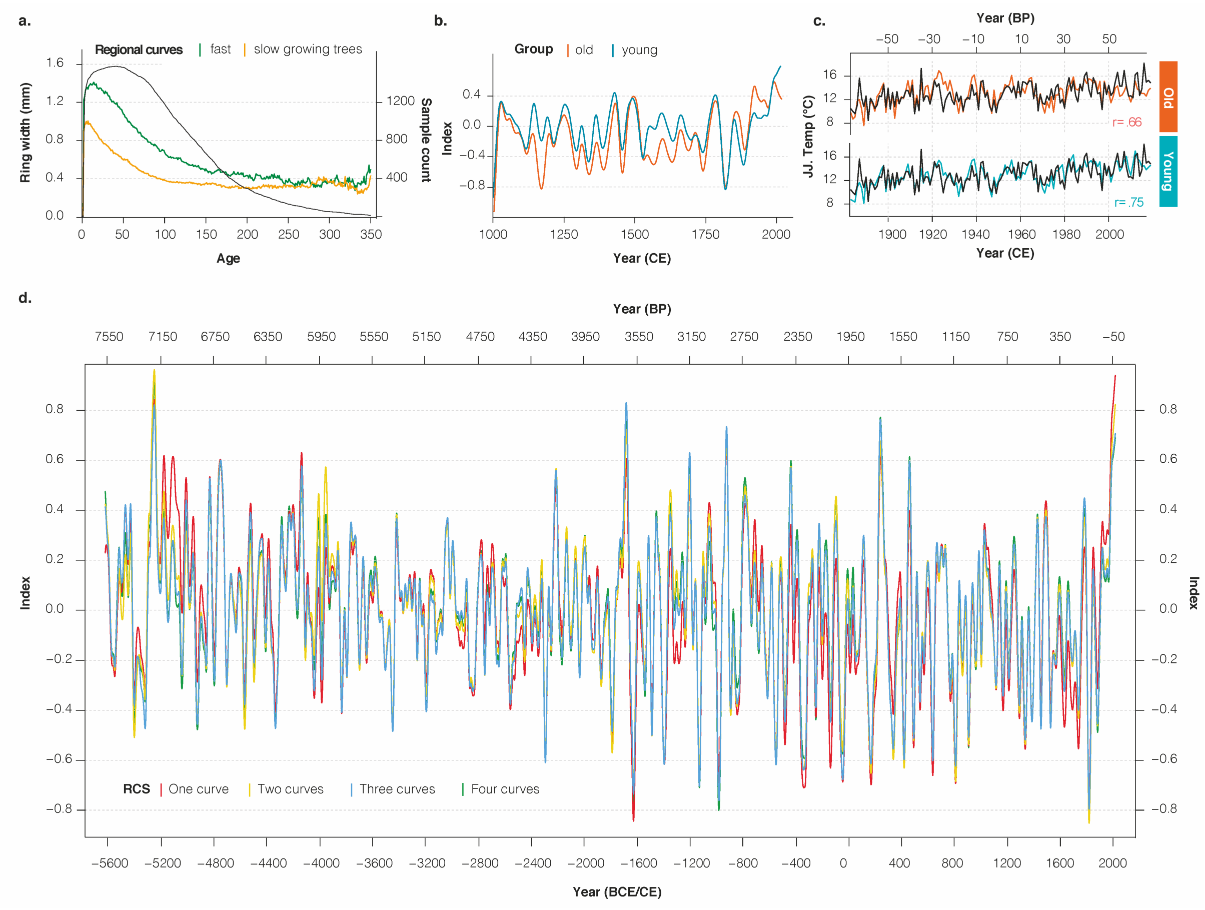

Supplementary figure 1. Signal Free RCS. a Regional curves for fast and slow growing trees with total sample count for each ring age. Trees have been assigned to the growing groups by assessing each tree's growth rate over the full life span of the tree relative to the growth rate of an unsmoothed SF-RC created using the measurement data from all trees[45, 46]. **b** Detrended and 50-yr cubic spline smoothed chronologies for young and old trees (i.e. below and above 100 years) over the last millennium. **c** Correlation of young and old trees chronologies with the 16 June to 4 August average temperature from the Salekard Meteorological station (black) over the period 1883-2019. **d** SF-RCS chronologies using 1 to 4 RCs applied to series grouped by their mean growth rates. Curves are normalized and smoothing with a 50-yr cubic spline. Moderate difference between the 1- and 2-curve SF-RCS chronologies indicates the need of

more curves to account for different growth rates associated with potential shifts in age distribution in the most recent period and that could lead to the modern sample bias[44]. Since the 2- and 3-curve SF-RCS chronologies are very similar, we applied the 2-curve SF-RCS for further analyses.

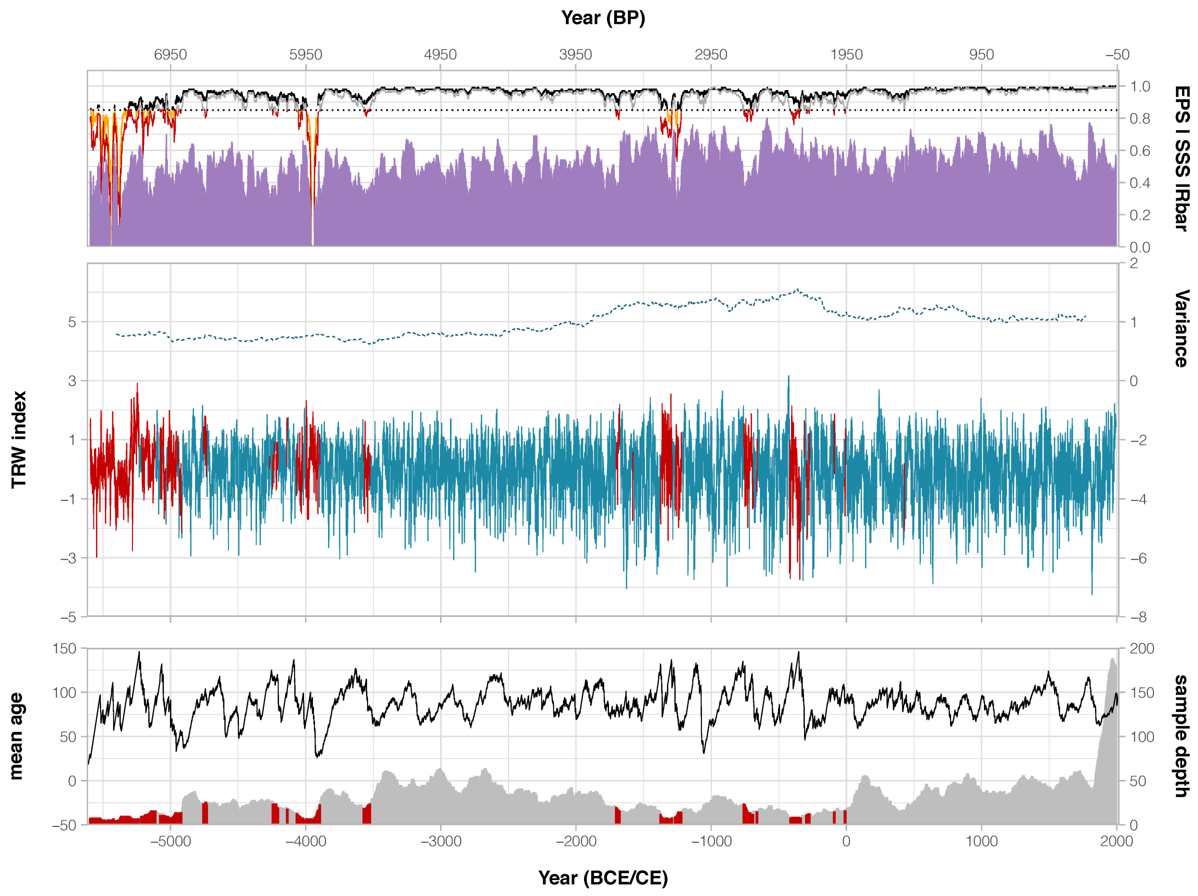

Supplementary figure 2. Chronology statistics of the Yamal7k chronology. a Expressed Population Signal adjusted to also represent long-timescale uncertainty (EPS; grey), Subsample Signal Strength (SSS; black), mean correlation among tree-ring series (Rbar; violet). **b** Tree-ring width index from the 2-curve SF-RCS detrended chronology (TRW index, blue) and its variance (dashed line). **c** Mean tree-ring age (Mean age; black), and sample depth (grey surface). EPS, SSS, and Rbar are calculated over 50-year windows, lagged by 25 years. Variance is calculated over 500-year windows, lagged by 1 year. Sample depth ranges from 4 to 187 series per year. Red/orange colors indicate periods when EPS<0.85.

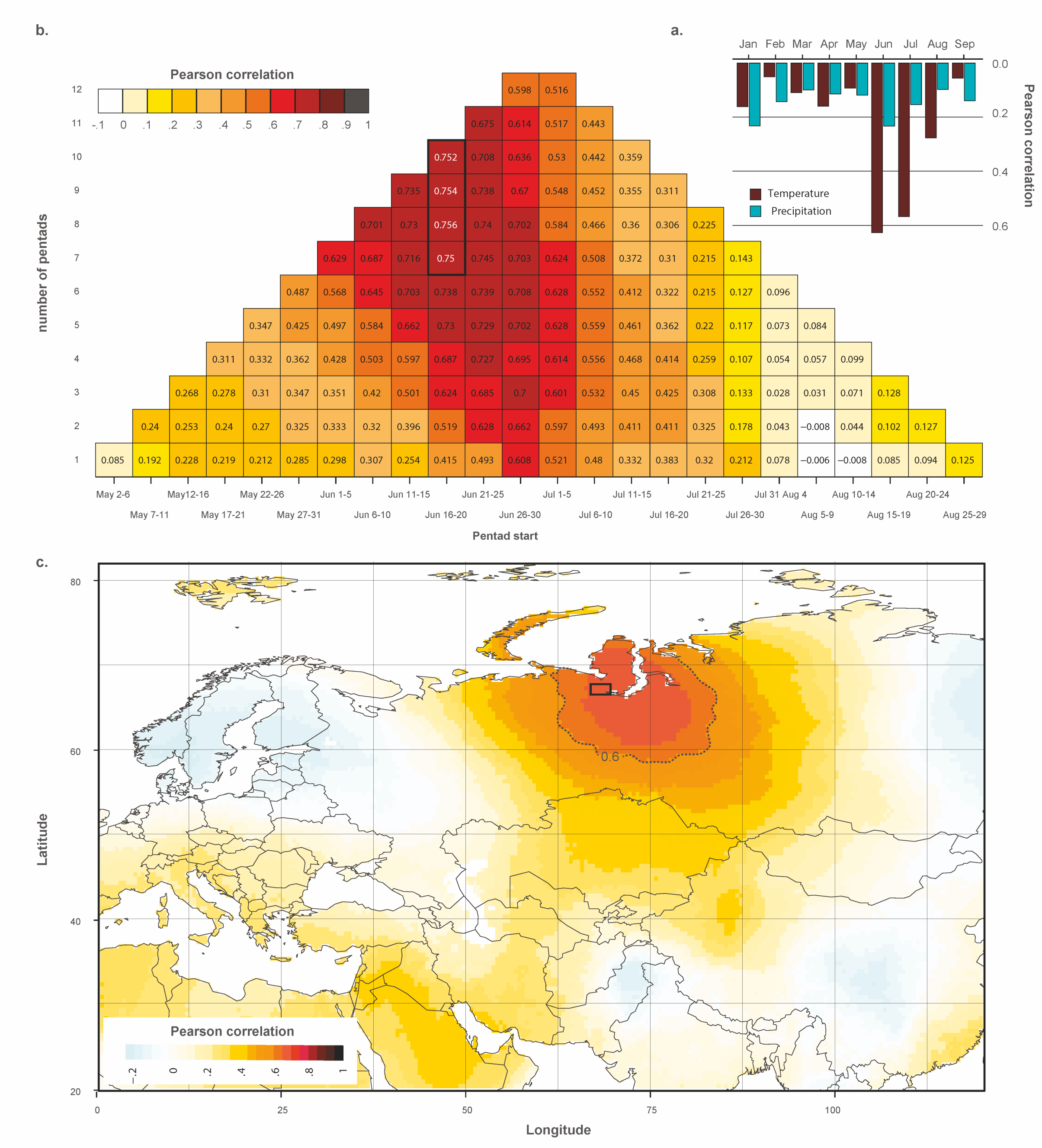

Supplementary figure 3. Correlation between the Yamal7k tree-ring chronology and climatic data. a Correlations between monthly temperature and precipitation from the Salekard Meteorological station and the Yamal7k tree-ring chronology over the period 1883-2019. **b** Correlation matrix between daily June-July temperatures from the Salekard meteorological station averaged over periods ranging from 5 to 60 consecutive days (1 to 12 pentads) and the Yamal 7k tree-ring chronology. **c** Correlation map between June-July 0.5°

gridded air temperatures (CRU TS4.04) and the Yamal7k tree-ring chronology over the common period 1901–2019 period. The dotted black line delimits the area for which correlations $r > 0.6$.

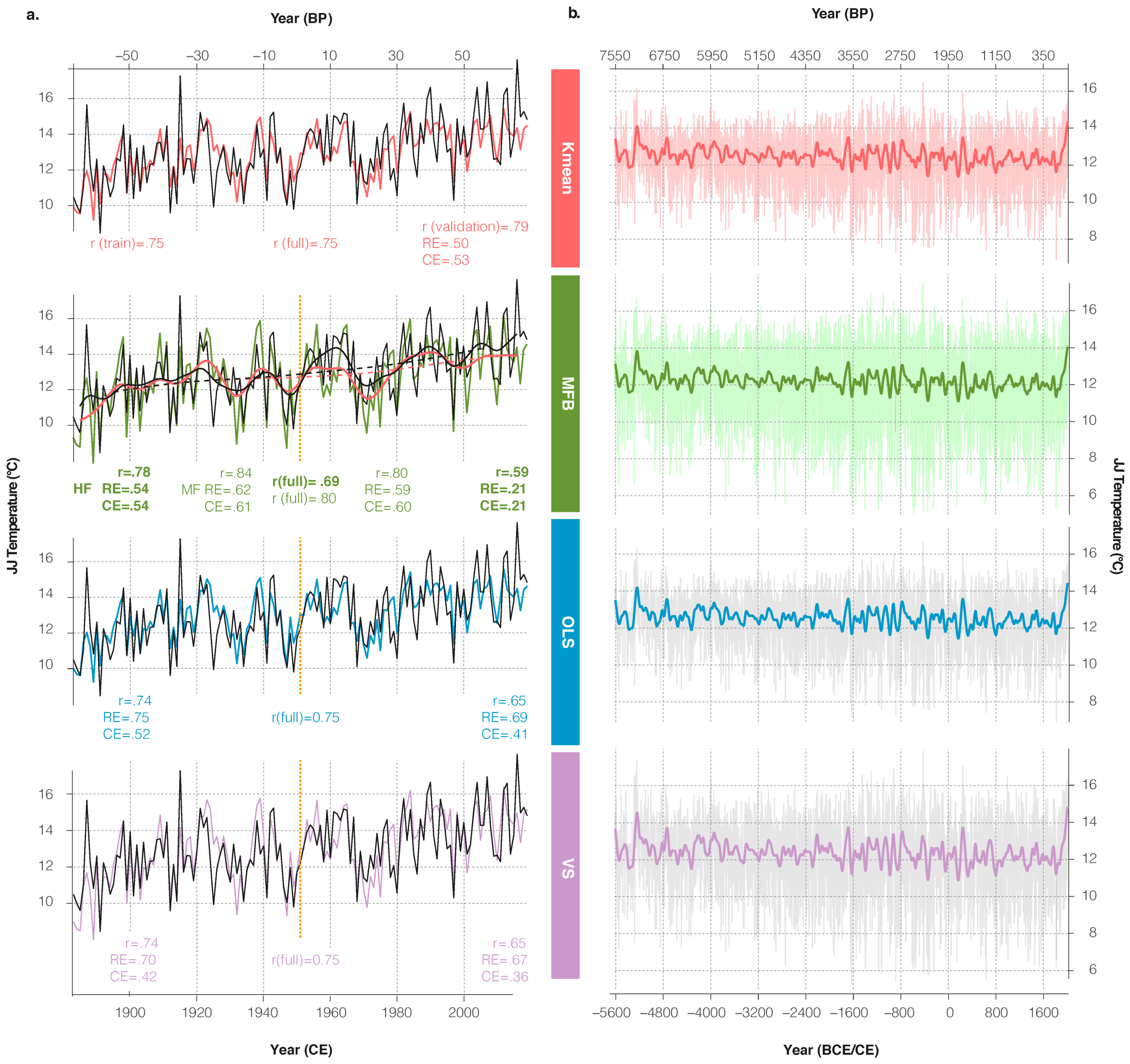

Supplementary figure 4. Overview of different June-July (JJ) temperature reconstructions and calibration-verification statistics. a Calibration-verification statistic based on ordinary least square (OLS), variance scaled (VS), multiple-frequency band (MFB) and K-fold cross-validation (Kmean) reconstructions with black lines indicating June 16 to August 4 mean temperatures as observed at Salekhard meteorological station. **b** June-July (JJ) temperature (5618 BCE to 2019 CE) for the four reconstructions. Thick lines are 100-year cubic spline smoothed reconstruction. Noteworthy, the calibration-verification statistics of the MFB reconstruction highlights a temperature-reconstruction comparison across multiple frequency bands, whereby dotted lines refer to low frequency (variability > 100 years), thin

lines indicate summed low and medium frequencies (MF between 15 and 100 years) and thick lines show summed low, medium and high frequencies (HF < 15 years). Minimum correlation between VS and the other three reconstructions over the 5618 BCE to 2019 CE period is 0.975.

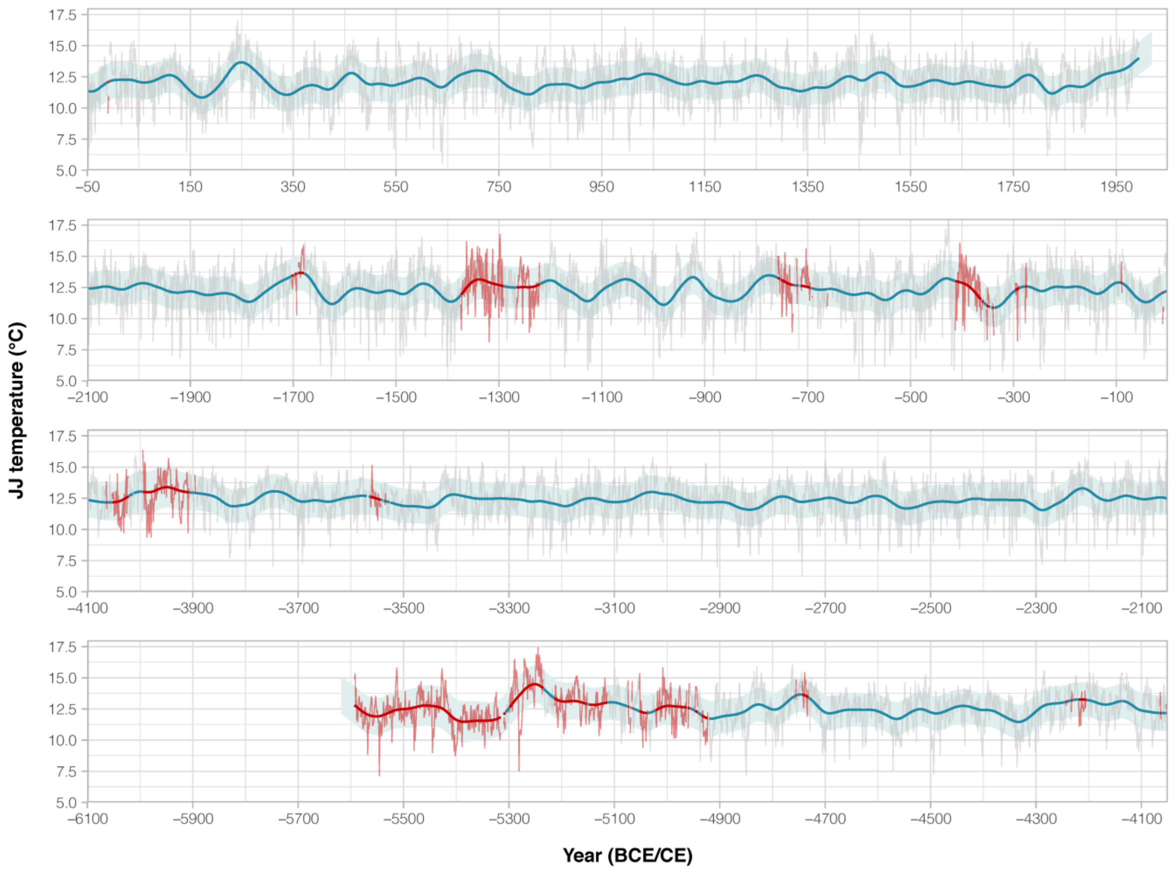

Supplementary figure 5. Reconstructed Yamal June-July temperature over the past 7,638 years. Variance scaled June-July temperature reconstruction plotted in four consecutive slices of 2 millennia. The grey line indicated the annually-resolved reconstruction. The blue line illustrates a 100-year cubic spline smoothed function to highlight mid-frequency climate variations at Yamal. The reconstruction uncertainty estimates (light blue band) incorporate both the ring-width chronology error and the reconstruction error (see Material and Methods for details). Red color indicates periods when EPS<0.85.

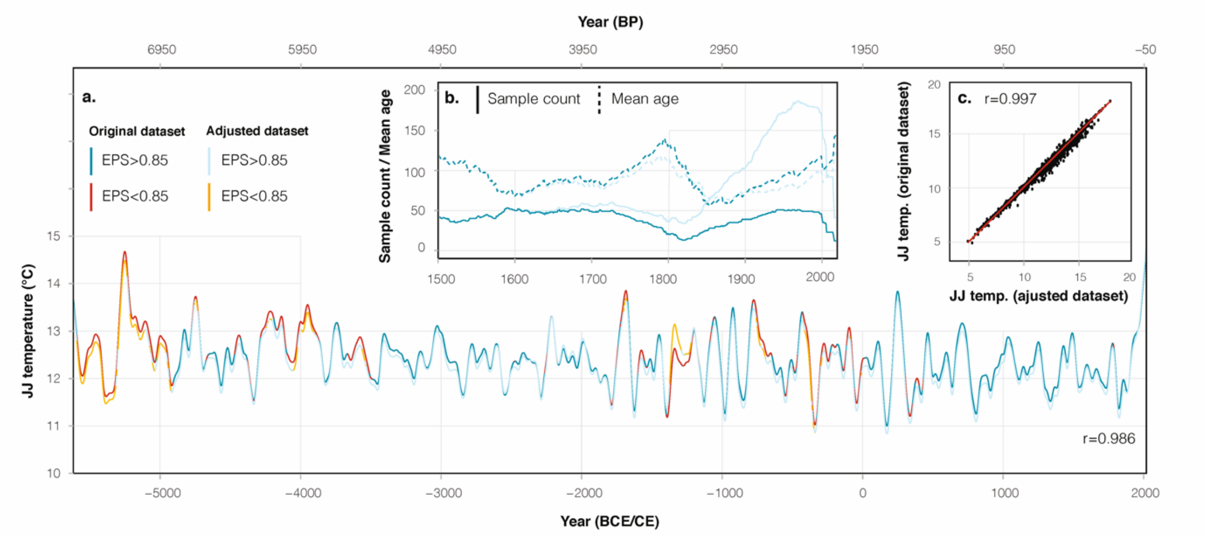

Supplementary figure 6. Comparison between original and subsampled Yamal7k JJ temperature reconstructions. a 100-years cubic splined smoothed JJ temperature reconstructions for the original and for the adjusted dataset where the sample count of the last century has been adjusted (by discarding samples at random) at a level consistent with the entire collection. **b** Sample count and mean age of both chronologies over the last 5 centuries. **c**. Correlation between original and adjusted JJ temperature reconstructions.

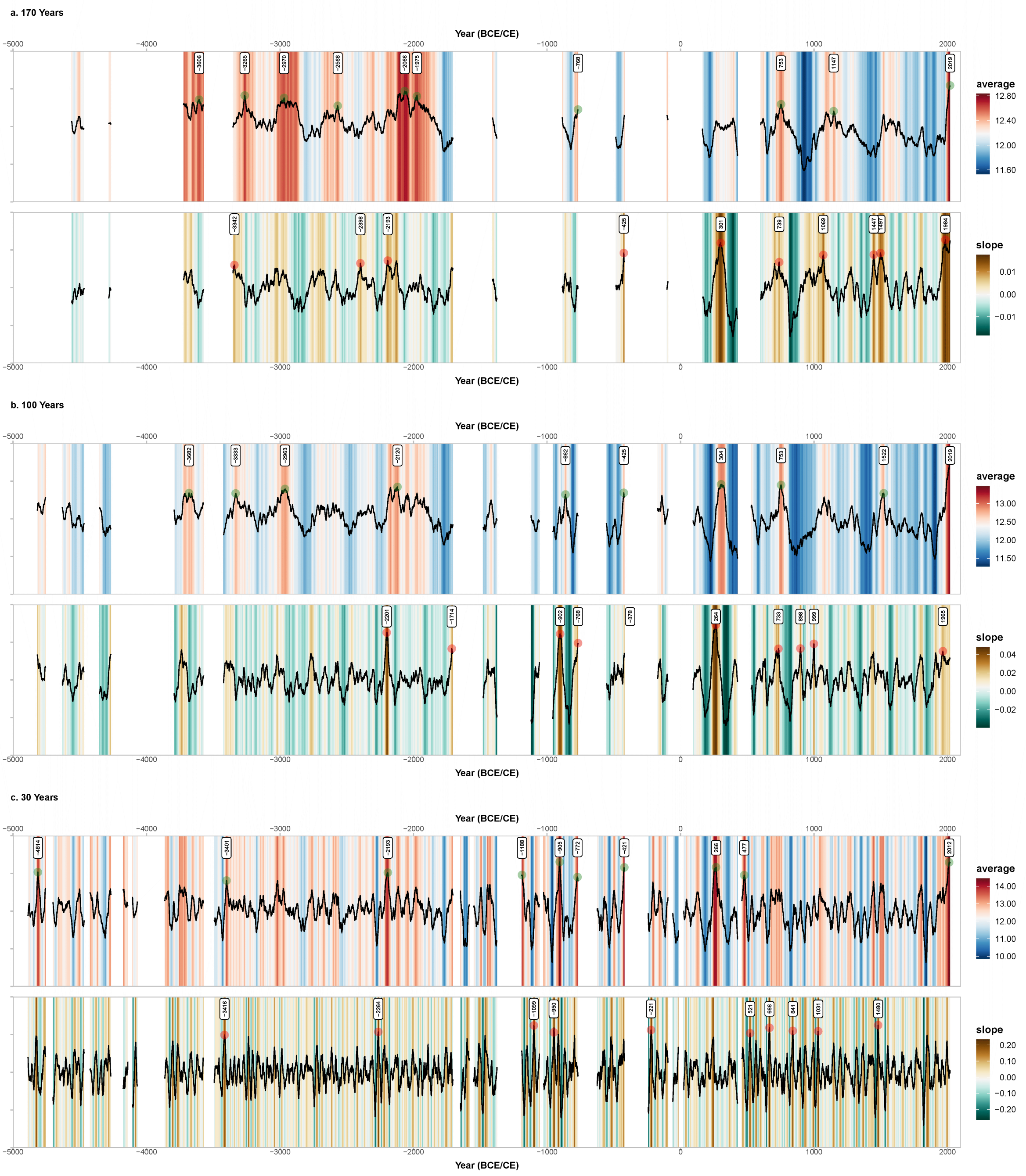

Supplementary figure 7. Average and slope variability. Plots of average and slope variability overlaid on stripe plots computed for timescales of 170, 100, and 30 years over the period -5000 BCE - 2019 CE. Labeled points indicate the timing of the right-centered periods with highest average and slopes. Time windows including years with EPS <0.85 are excluded from the analyses.

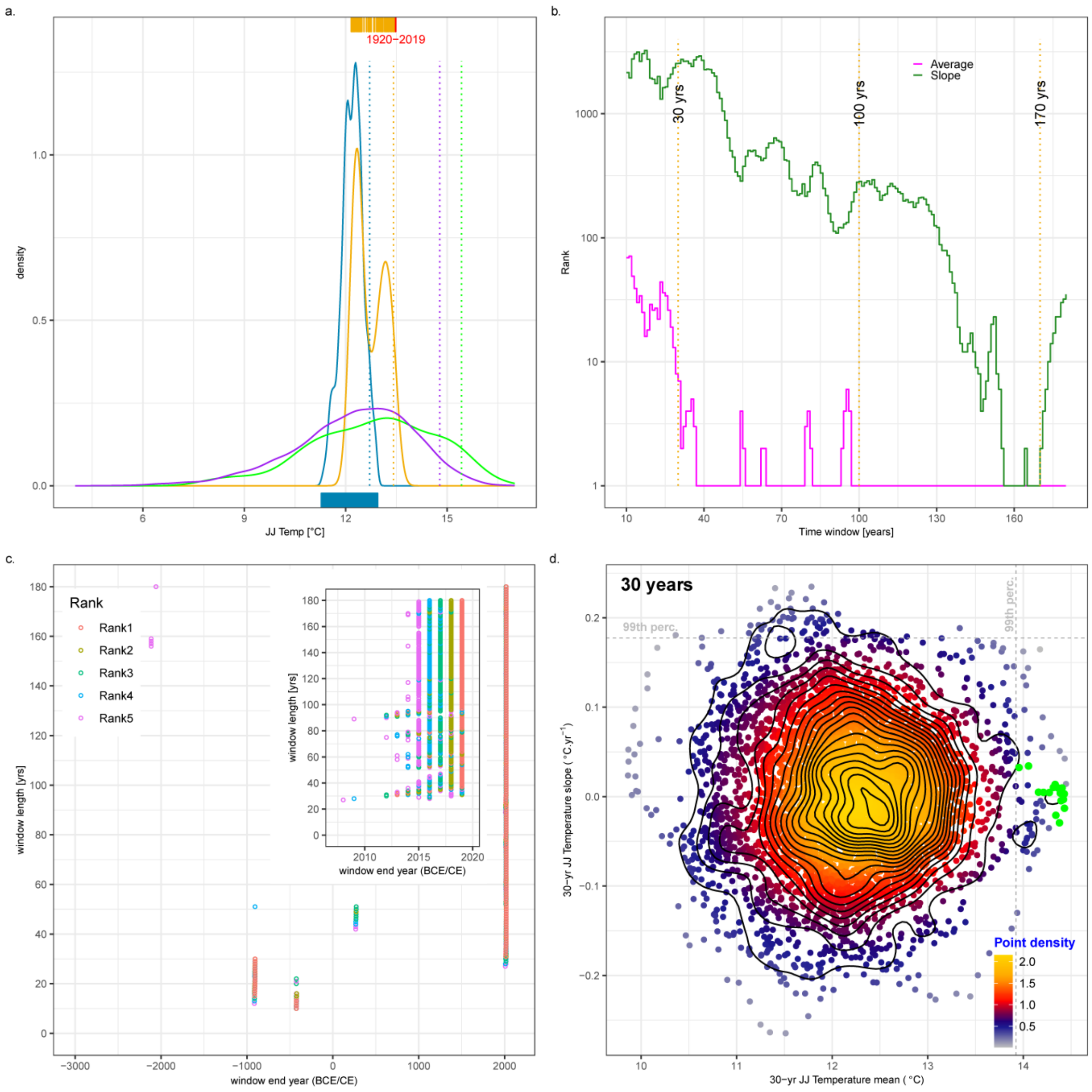

Supplementary figure 8. Distribution and ranking of JJ temperature (average and rate) in the Yamal Peninsula over the 5618 BCE to 2019 CE period. a Kernel distributions of individual yearly JJ temperatures split into two periods (purple: 5618 BCE – 1850 CE, and green: 1851-2019 CE) and of 100-year means of JJ temperatures for two periods (blue: 100-year means ending before 1850; orange: 100-year means ending after 1850). Dotted lines indicate the respective 95th percentiles of the distributions. Vertical lines at the top (orange and red for the 100-yr mean 1920-2019) and bottom (blue) of the graph indicates the individual

values for each of the 100-yr means. **b** Ranks of June-July temperature averages (magenta) and slopes (green) computed over time windows ranging from 10 and 180 years and all ending in 2019 CE. **c** Overview of the warmest five (Rank 1 to 5) periods (x-axis, with the date indicating the end of the window length used to compute June-July temperature average and slope) as a function of period length (y-axis). The inset provides details over the warmest periods ending between 2005 and 2019 CE. **d** Two-dimensional density plots of temperature trends versus mean temperatures computed for 30-year time windows. Green dots indicate the 30 years periods ending after 2002 CE.

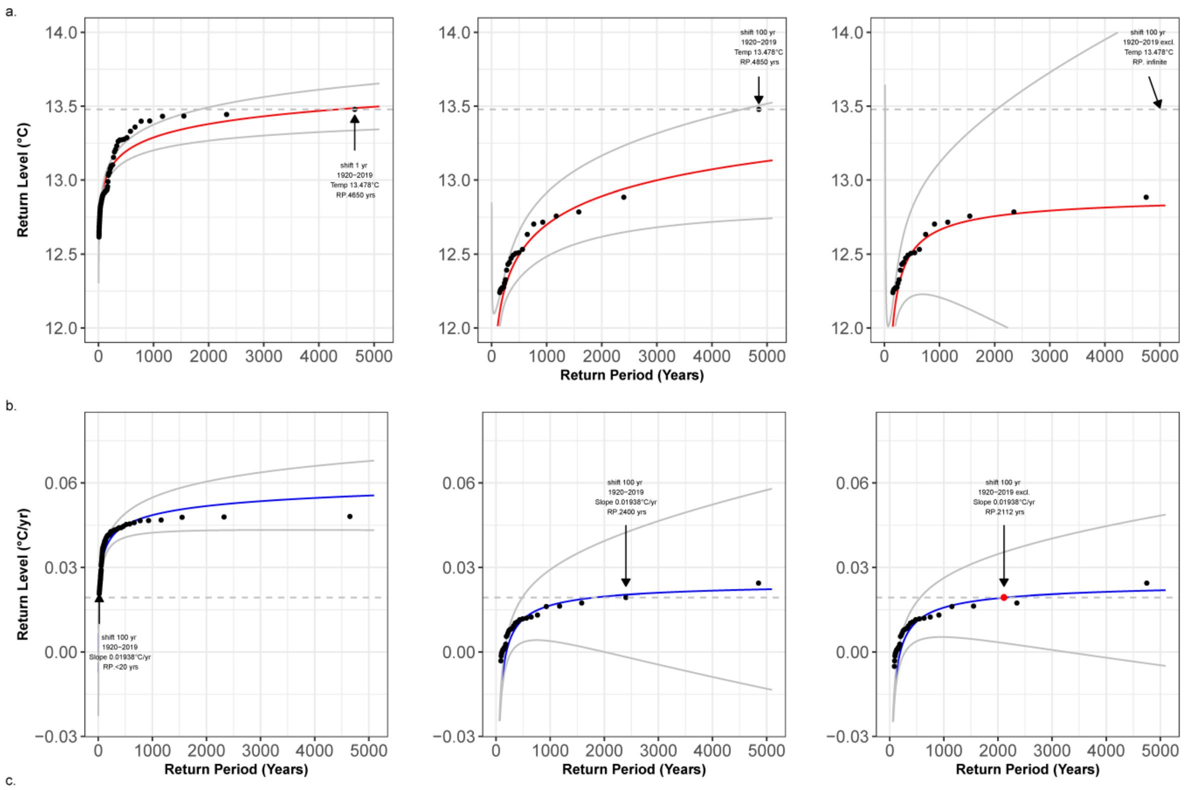

c.

Time window	Reference period	Shift	Ref. period included	Parameter	Threshold (percentile)	Nb values >th	Param. Value	Return period
30 yr	1990-2019	1 yr	yes	Average	0.95	284	14.405 °C	810 yrs
30 yr	1990-2019	30 yr	yes	Average	0.5	95	14.405 °C	5715 yrs
30 yr	1990-2019	30 yr	no	Average	0.5	94	14.405 °C	Infinite
30 yr	1990-2019	1 yr	yes	Slope	0.95	284	0.0097 °C/yr	<20 yrs
30 yr	1990-2019	30 yr	yes	Slope	0.5	95	0.0097 °C/yr	74 yrs
30 yr	1990-2019	30 yr	no	Slope	0.5	94	0.0097 °C/yr	74 yrs
100 yr	1920-2019	1 yr	yes	Average	0.9	465	13.478 °C	4650 yrs
100 yr	1920-2019	100 yr	yes	Average	0.5	24	13.478 °C	4850 yrs
100 yr	1920-2019	100 yr	no	Average	0.5	23	13.478 °C	Infinite
100 yr	1920-2019	1 yr	yes	Slope	0.95	233	0.0193 °/yr	<20 yrs
100 yr	1920-2019	100 yr	yes	Slope	0.3	33	0.0193 °/yr	2400 yrs
100 yr	1920-2019	100 yr	no	Slope	0.3	32	0.0193 °/yr	2112 yrs
170 yr	1850-2019	1 yr	yes	Average	0.95	192	12.833 °C	3824 yrs
170 yr	1850-2019	170 yr	yes	Average	0.1	21	12.833 °C	4224 yrs
170 yr	1850-2019	170 yr	no	Average	0.1	20	12.833 °C	Infinite
170 yr	1850-2019	1 yr	yes	Slope	0.95	192	0.0173 °C/yr	1912 yrs
170 yr	1850-2019	170 yr	yes	Slope	0.1	21	0.0173 °C/yr	4224 yrs
170 yr	1850-2019	170 yr	no	Slope	0.1	20	0.0173 °C/yr	Infinite

Supplementary figure 9. Return periods for 1990-2019, 1920-2019 and 1850-2019 JJ Temperature Average and Slope based on the Generalized Pareto Distribution (GPD). **a-b** Return period estimated for the 1920-2019 reference period for mean temperature (Average) (**a**) and warming rate (Slope) (**b**). On the left column, centennial temperatures have been computed using windows shifted by steps of 1-year (e.g. 1920-2019, 1919-2018, 1918-2017, …) and by 100-years moving windows for the central and right columns (e.g. 1920-2019, 1820-1919, 1720-1918, …). Right column is similar to the central column with the

1920-2019 period excluded from the GPD fitting. **c** Synthesis of the return period obtained for the different periods (30, 100 and 170 yrs) and time window shifts (1, 30, 100, and 170 yrs). Time windows including years with EPS <0.85 are excluded from the analyses.

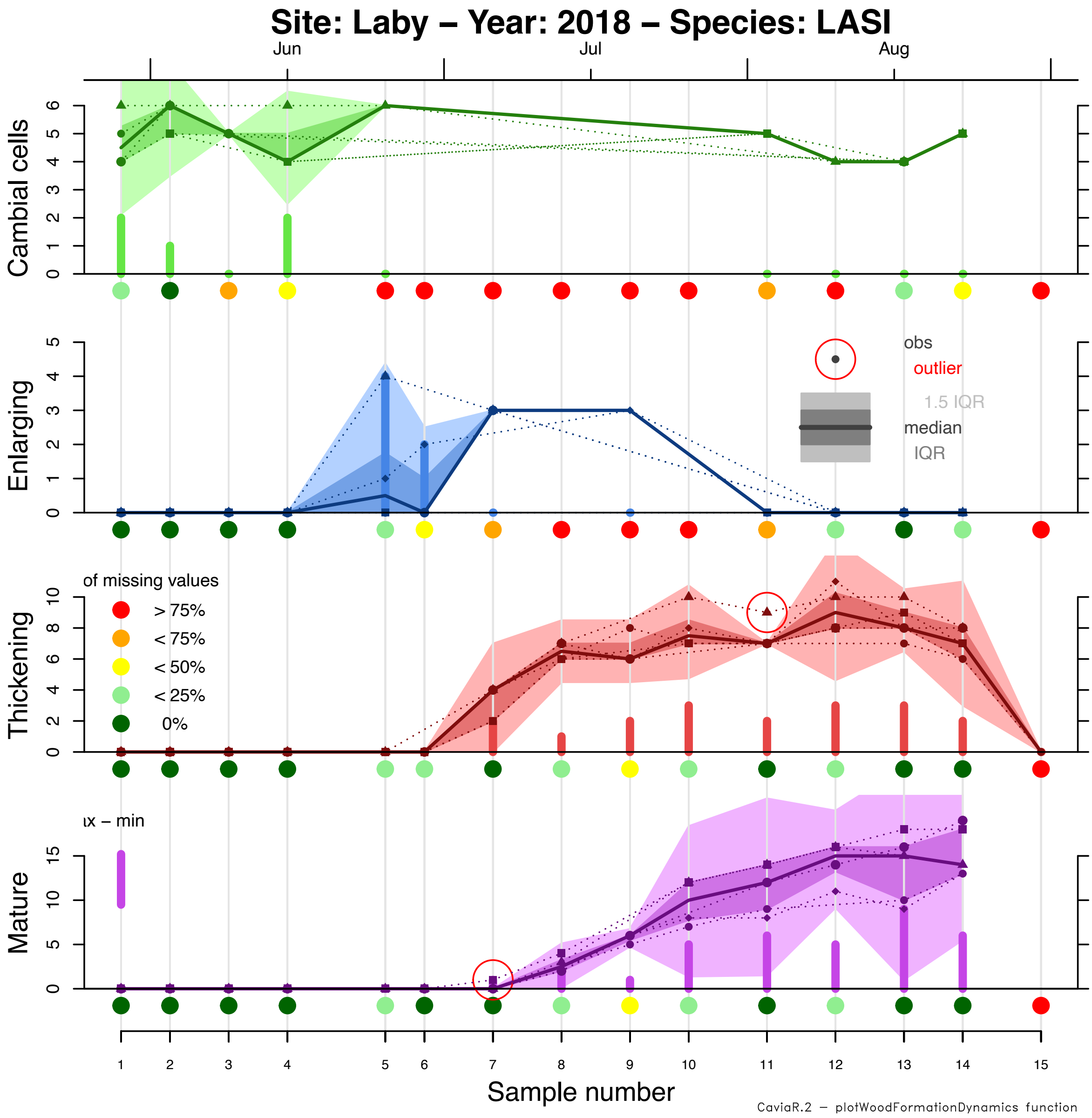

Supplementary figure 10. Dynamic of tree-ring formation as observed during the 2018 growing season (May 29th to August 31st) on five *Larix sibirica* individuals near Salekhard. The thick lines indicate the medians of the developing tracheids (cambial, enlarging, thickening and mature cells) counted along three radial files in the forming ring. The points connected with dotted lines indicate the radial file raw data, the dark envelopes indicate inter-quartile range (IQR); the light envelopes, 1.5 × IQR; and the red circles, outliers outside 3 × IQR. The color of the dots under the *x*-axis indicates the level of missing values. The vertical bars show the maximum range between raw data for a sample.

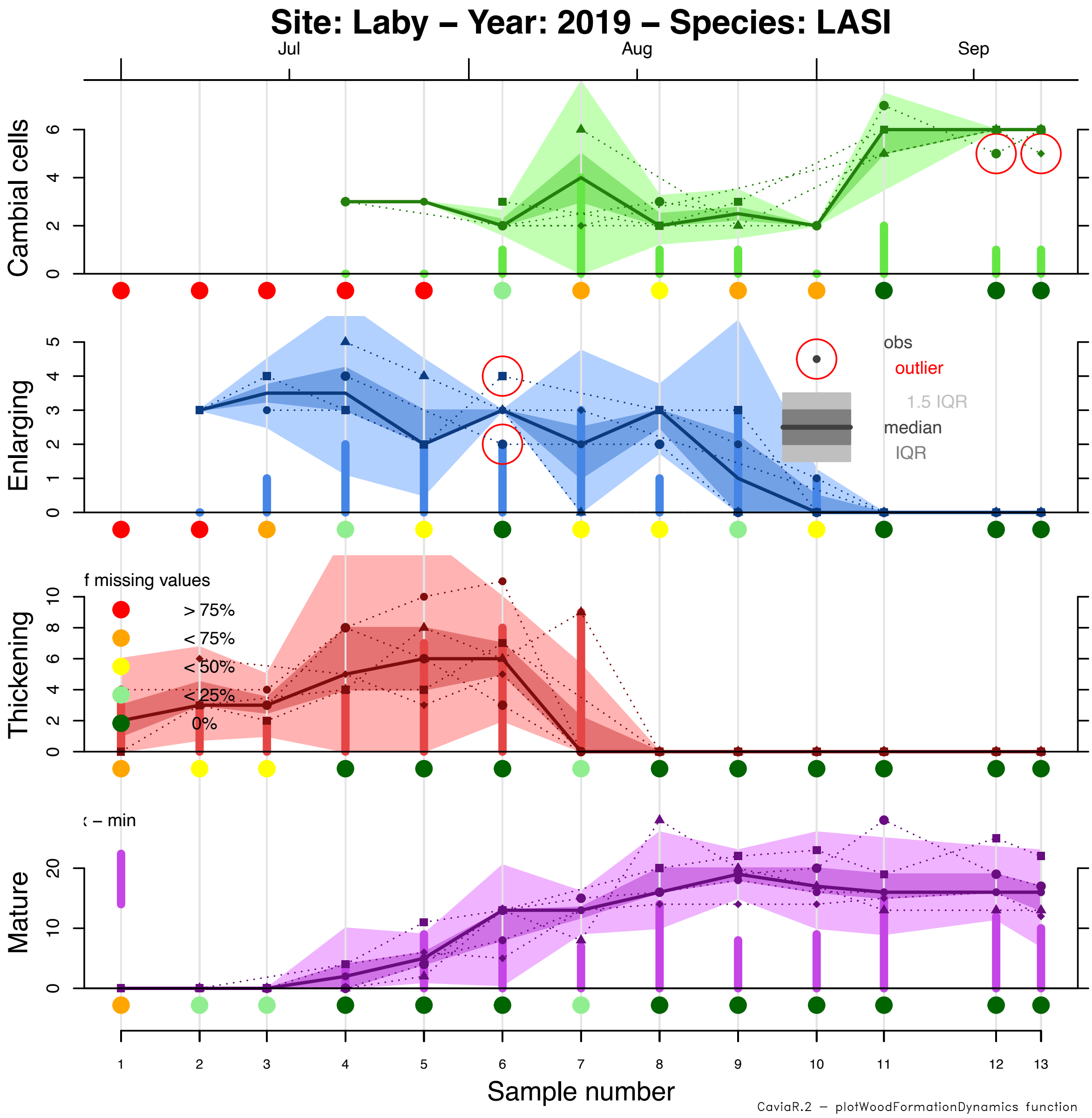

Supplementary figure 11. Dynamic of tree-ring formation as observed during the 2019 growing season (July 1st to September 21st) on five *Larix sibirica* individuals near Salekhard. The thick lines indicate the medians of the developing tracheids (cambial, enlarging, thickening and mature cells) counted along three radial files in the forming ring. The points connected with dotted lines indicate the radial file raw data, the dark envelopes indicate inter-quartile range (IQR); the light envelopes, 1.5 × IQR; and the red circles, outliers outside 3 × IQR. The color of the dots under the *x*-axis indicates the level of missing values. The vertical bars show the maximum range between raw data for a sample.

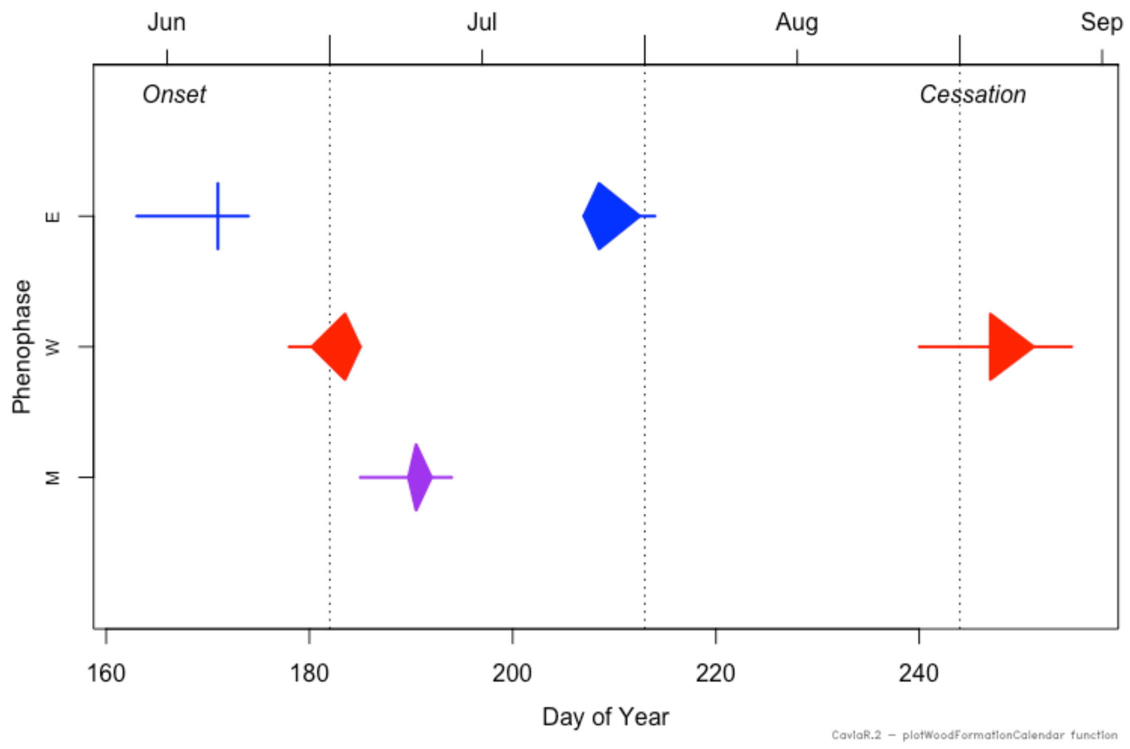

Supplementary figure 12. Wood formation calendar as observed during the 2018-2019 growing seasons on five *Larix sibirica* individuals near Salekhard. The plot indicates the critical dates and durations for each phenophase: E = enlargement (in blue, indicating also the period of radial stem growth); W = cell wall thickening (in red, indicating also the period of mass growth); and M = mature cells (in purple). Crossed diamond shape signs show minimal, second quartile, median, third quartile and group maximum values.

SUPPLEMENTARY TABLE

a

Method	Calibration Period	r	r²	Durbin Watson	EVE	% Variance loss	Verification Period	r	r²	RE	CE
**OLS**	1883-1951	0.74	0.55	1.76			1952-2019	0.65	0.43	0.69	0.41
	1952-2019	0.65	0.43	1.87			1883-1951	0.74	0.55	0.75	0.52
	1883-2019	0.75	0.57	1.68							
**VS**	1883-1951	0.74	0.55		0.48	-12.52	1952-2019	0.65	0.43	0.67	0.36
	1952-2019	0.65	0.43		0.31	18.41	1883-1951	0.74	0.55	0.70	0.42
	1883-2019	0.75	0.57		0.50	-10.87					
**MFB-HF**	1885-1951	0.78	0.60		0.55	-8.32	1952-2017	0.59	0.35	0.21	0.21
	1952-2017	0.59	0.35		0.18	10.85	1885-1951	0.78	0.60	0.54	0.54
	1885-2017	0.69	0.48		0.38	-19.72					
**MFB-MF**	1896-1951	0.84	0.71		0.69	-3.44	1952-2006	0.80	0.63	0.60	0.59
	1952-2006	0.80	0.63		0.59	-5.06	1896-1951	0.84	0.71	0.62	0.61
	1896-2006	0.80	0.64		0.60	-6.22					

b

Method	Train data	r	r²	Durbin Watson	Train model	Evaluation data	r	r²	RE	CE
**Kmean**	80%	0.75	0.57	1.69	T = 1.290734x + 12.405331	20%	0.79	0.62	0.55	0.53

Supplementary Table 1. Calibration-verification statistics of the four reconstructions. a Statistics of the full (1883–2019) calibration model, as well as using two equally-long early/late (1883–1951 and 1952–2019) split period calibration windows for each of the three calibration methods applied. **b** Statistics of the *K*-fold cross-validation[50] (calculated to test the reconstruction function using a binary linear regression model based on least squares over the full observation period 1883–2019). To implement the *K*-fold approach, the dataset was randomly partitioned into *K* equal-sized sub-datasets (known as folds). *K*-1 of these folds were used to train the function on 80% of the data, and the remaining fold was used for testing as evaluation data. This procedure was executed *K*=10 times, with each fold removed in turn for testing. The results from the *K*-folds were averaged together to get a final result for the reconstruction function. OLS = Ordinary least square; VS = variance scaled; MFB-HF = High-Frequency (i.e. variability changes occurring over period less than 15 years) from the Multiple frequency band; MFB-MF = Medium-Frequency (i.e. variability changes occurring between

15 and 100 years) from the Multiple frequency band; Kmean = *K*-fold cross-validation; r = Pearson correlation; r^2 = Coefficient of determination; EVE = Equivalent Variance explained; RE = reduction of error; CE= Coefficient of efficiency. Autocorrelation over the period 1883-2019 is 0.249 for the instrumental data, 0.302 for MFB (all frequencies), and 0.533 for the reconstructions.
